# Autoantibodies against neuronal surface proteins in spontaneous subarachnoid and intracerebral haemorrhage

**DOI:** 10.1186/s12883-018-1097-1

**Published:** 2018-06-28

**Authors:** Harald Hegen, Raimund Helbok, Mario Kofler, Bettina Pfausler, Alois Schiefecker, Erich Schmutzhard, Ronny Beer

**Affiliations:** 1 Department of Neurology, Medical University of Innsbruck, Innsbruck, Austria; 2 Neurological Intensive Care Unit, Department of Neurology, Medical University of Innsbruck, Anichstrasse 35, A-6020 Innsbruck, Austria

**Keywords:** Subarachnoid haemorrhage, Intracerebral haemorrhage, Immunoreactivity, Neuronal surface proteins, NMDA receptor, GABA_B_ receptor, AMPA receptor, LGI-1, CASPR-2

## Abstract

**Background:**

Brain autoimmunity has been reported in patients with preceding infection of the central nervous system by *herpesviridae*. It has been hypothesized that neuronal damage releasing antigens might trigger secondary immune response. The objective of the study was to investigate whether brain damage due to spontaneous subarachnoid haemorrhage (SAH) or intracerebral haemorrhage (ICH) induces reactivity against neuronal surface proteins.

**Methods:**

Patients with spontaneous SAH and ICH, who had cerebrospinal fluid (CSF) and serum sampling within 2 weeks after disease onset (baseline) and afterwards at least 10 days later (follow-up), were included. Antibodies against NMDA, GABA-B, AMPA-1/− 2 receptor, LGI1 and CASPR2 were determined by indirect immunofluorescence.

**Results:**

A total of 43 SAH and 11 ICH patients aged 62 (±12) years (65% females) had simultaneous CSF/ serum sampling median 5 and 26.5 days after disease onset. At baseline, all CSF samples were collected via ventricular drainage, at follow-up 20 (37.0%) patients had CSF collection by lumbar puncture because ventricular drain had been already removed. All CSF and serum samples at baseline and follow-up tested negative for antibodies against NMDA, GABA-B, AMPA-1/− 2 receptor, LGI1 and CASPR2.

**Conclusions:**

Immunoreactivity against common neuronal surface proteins was not observed within the early disease course of spontaneous SAH and ICH.

## Background

Spontaneous subarachnoid haemorrhage (SAH) and spontaneous intracerebral haemorrhage (ICH) are both severe neurological disorders that result in brain injury due to several mechanisms including primary mechanical tissue damage caused by the bleeding and secondary effects such as inflammatory processes, oxidative stress responses, brain edema and in case of SAH delayed cerebral ischemia [1, 2].

With cell injury and blood brain barrier disruption antigens are released into the intrathecal space and, therefore, abundantly presented to the immune system. This may result in breaking tolerance and in the formation of an immune response. Brain autoimmunity has been observed in patients with preceding infections of the central nervous system (CNS) by *herpesviridae* [3–8]. In these patients, antibodies against N-methyl-D-aspartate (NMDA) receptor and other, so far unknown neuronal surface antigens, appeared between one to several weeks after disease onset [3]. It has been hypothesized that antigens being released through neuronal damage trigger secondary immune reactivity [9].

In the present study, we aimed to investigate whether acute brain damage due to spontaneous SAH or ICH induces the formation of antibodies against neuronal surface proteins in the early and post-acute phase.

## Methods

### Patients and samples

**Fig. 1:**
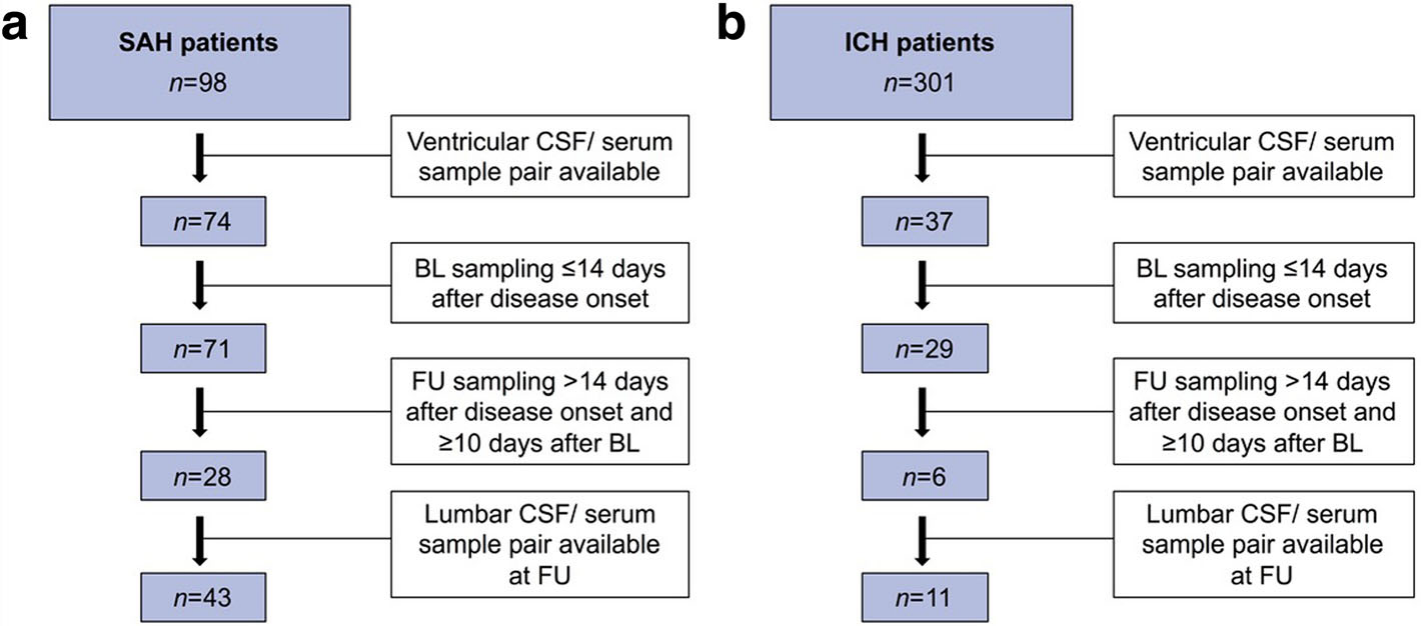
Sample Flow Chart. **a** Subarachnoid haemorrhage patient cohort; **b** Intracerebral haemorrhage patient cohort. A total of 43 SAH and 11 ICH patients had simultaneous CSF and serum sampling at baseline and at follow-up

Patients with spontaneous SAH and ICH admitted within 24 h of disease onset to the neuro-intensive care unit of Medical University of Innsbruck have been recorded in a computerized database since April 2010 and July 2011, respectively. Diagnoses were confirmed by computed tomography of the head and traumatic causes were excluded. Only patients who had ventricular cerebrospinal fluid (CSF) and serum sampling within 2 weeks after disease onset (baseline) and afterwards at least 10 days later (follow-up), were included into this retrospective analysis. At follow-up, also lumbar CSF analysis was eligible in case of lacking ventricular CSF sampling (Fig. 1). All CSF and serum samples were withdrawn simultaneously and stored at − 20 °C until analysis.

### Immunofluorescence assay

Antibodies against NMDA, γ-aminobutyric acid type B (GABA_B_), α-amino-3-hydroxy-5-methyl-4-isoxazolepropionic acid (AMPA)-1, AMPA-2 receptor and against the voltage-gated potassium channel (VGKC)-associated proteins leucine-rich glioma inactivated 1 (LGI1) and contactin-associated protein 2 (CASPR2) were determined in CSF and serum at baseline and follow-up by commercially available cell-based indirect immunofluorescence assays (Cat.No. FA112d-1010-51, FA112l-1005-50, FA112k-1005-1 and FA1439–1005-1; Euroimmun, Lübeck, Germany).

Briefly, CSF and serum samples were added to an object plate containing human embryonic kidney cells, which express the respective antigen on their surface. After incubation, detection of patients’ autoantibodies was achieved by fluorescein isothiocyanate-conjugated anti-human immunoglobulin G antibodies that were finally visualized by fluorescence microscopy.

### Statistical analysis

We performed statistical analysis using SPSS 23.0 (SPSS Inc., Chicago, IL). Distribution of data was assessed by Kolmogorov-Smirnov test and, accordingly, data were displayed as mean ± standard deviation (SD) or as median and interquartile range (IQR).

## Results

A total of 43 SAH and 11 ICH patients aged 62 (±12) years comprising 65% females had simultaneous CSF and serum sampling at baseline, median 5 (IQR 3–8) days after disease onset, and at follow-up after 26.5 (19–35.5) days. At baseline, all CSF samples were collected via ventricular drainage. At follow-up, 15 (34.9%) SAH and 5 (45.5%) ICH patients had CSF collection by lumbar puncture because ventricular drain had been already removed. Demographic and main sample characteristics for each disease group are displayed in Table 1.

**Table 1:** Demographic data and cerebrospinal fluid characteristics at baseline and follow-up

	SAH	ICH
n	43	11
Age, years; mean ± SD (range)	60.7 ± 12.2 (28–86)	67.6 ± 9.3 (55–85)
Sex, female; n (%)	29 (67.4)	6 (54.5)
Hunt & Hess, n (%)		
Grad I-II	6 (14.0)	
Grad III	12 (27.9)	
Grad IV-V	25 (58.1)	
Time between disease onset and BL sampling, days	5 (3–8)	6 (4–9)
Time between disease onset and FU sampling, days	27 (19–39)	26 (19–33)
Baseline CSF findings		
RBC, /μl	32,000 (10667–96,000)	30,000 (3093–130,000)
WBC, /μl	91 (28–371)	73 (35–363)
Total protein, mg/l	1910 (880–2870)	2520 (1040–4280)
FU CSF findings		
RBC count, /μl	106 (13–419)	72 (3–3072)
WBC count, /μl	9 (2–18)	18 (10–36)
Total protein, mg/l	410 (320–690)	840 (445–1625)

Data are given as median (interquartile range) unless otherwise specified

*BL* baseline, *CSF* cerebrospinal fluid, *FU* follow-up, *ICH* intracerebral haemorrhage, *RBC* red blood cell, *SAH* subarachnoid haemorrhage, *WBC* white blood cell

### Negative antibodies against neuronal surface proteins

All CSF and serum samples at baseline and follow-up were tested negative for antibodies against NMDA, GABA_B_, AMPA-1, AMPA-2 receptor and against the VGKC-associated proteins LGI1 and CASPR2.

## Discussion

According to recent reports on patients with CNS infection by *herpesviridae* who developed secondary brain autoimmunity as shown by the detection of anti-NMDA receptor antibodies and other so far unknown neuronal surface antibodies within weeks after disease onset [3–8], one might hypothesize that neuronal damage in other CNS disorders might also result in the formation of a secondary immune response. In the present study, we screened for antibodies against NMDA, GABA_B_, AMPA-1, AMPA-2 receptors and against the VGKC-associated proteins LGI1 and CASPR2 in the CSF and serum of patients with spontaneous SAH and ICH. Within a median time period of approximately 4 weeks after disease onset, we did not obtain any positive result.

There might be several reasons for the absence of a secondary immune response in our cohort of SAH and ICH patients. First, the aetiology of the evolution of anti-NMDA receptor antibodies in patients with preceding *herpesviridae* encephalitis is still unknown [9]. Although it has been hypothesized that a release of antigens by viral neuronal cell lysis triggers an immune response that is misdirected against a structurally similar epitope present in the NMDA receptor [9], there might be other or additional, so far unknown factors required for the initiation of CNS autoimmunity that are lacking or altered in patients with intrathecal bleeding. Secondly, immune reactivity with detectable levels of autoantibodies could start after the observation period of our patients (in our cohort only 10 out of 54 patients had follow-up sample later than 6 weeks).

There are some limitations of the study, e.g. the small number of patients. One could argue that autoantibodies against neuronal surface proteins might occur at a low frequency, and, therefore, were not detected. Although a higher patient number would strengthen the (negative) results, one has still to consider a false positive rate of the immunofluorescence test of up to 1% (as determined in healthy individuals) [10, 11]. Furthermore, as only a small proportion of the entire SAH and ICH cohort was eligible for determination of neuronal surface antibodies depending on availability of CSF and serum samples, it cannot be excluded that a selection bias has been introduced. Even though patients requiring ventricular drainage usually present with a more severe disease course, i.e. show more extensive brain damage, that possibly predisposes to secondary immune response formation, these patients typically show more intrathecal blood. It is unclear whether the presence of intrathecal blood might hamper the local establishment of a specific immune reactivity due to its potential ability of blocking epitopes.

Finally, antibodies against neuronal surface proteins cover only a small part of possible antigens against an immune response can be directed. Therefore, screening for antibodies against neuropil as well as determination of antibodies against intracellular epitopes has to be done in a further study.

## Conclusions

Immunoreactivity against common neuronal surface proteins was not observed within the early disease course of spontaneous SAH and ICH.

## Abbreviations

AMPA: α-amino-3-hydroxy-5-methyl-4-isoxazolepropionic acid
CASPR2: contactin-associated protein 2
CNS: central nervous system
CSF: cerebrospinal fluid
GABA_B_: γ-aminobutyric acid type B
ICH: intracerebral haemorrhage
LGI1: leucine-rich glioma inactivated 1
NMDA: N-methyl-D-aspartate
SAH: subarachnoid haemorrhage
VGKC: voltage-gated potassium channel
